# Analysis of the hormone receptor status of circulating tumor cell subpopulations based on epithelial-mesenchymal transition: a proof-of-principle study on the heterogeneity of circulating tumor cells

**DOI:** 10.18632/oncotarget.11787

**Published:** 2016-09-01

**Authors:** Xiuwen Guan, Fei Ma, Suyan Liu, Shiyang Wu, Rong Xiao, Lifang Yuan, Xiaoying Sun, Zongbi Yi, Huiyi Yang, Binghe Xu

**Affiliations:** ^1^ Department of Medical Oncology, Cancer Hospital, Chinese Academy of Medical Sciences and Peking Union Medical College, Beijing, China; ^2^ SurExam Bio-Tech, Guangzhou Technology Innovation Base, Science City, China; ^3^ Department of Medical Oncology, Huanxing Cancer Hospital, Beijing, China

**Keywords:** breast cancer, circulating tumor cells, epithelial-mesenchymal transition, hormone receptor, subpopulations

## Abstract

Although the enumeration of circulating tumor cells (CTCs) has been demonstrated to be a prognostic indicator in metastatic breast cancer, the heterogeneous characteristics of CTCs, such as variations in the epithelial-mesenchymal transition (EMT), may limit its broad clinical application. To investigate an uncomplicated and practicable detection approach based on the potential utility of the heterogeneity of CTCs from the standpoint of the EMT phenotype and ER/PR status of CTCs, an analysis was conducted using peripheral blood samples obtained from 28 metastatic breast cancer patients. The CanPatrol CTC enrichment technique was used to identify different CTC subpopulations, including epithelial-dominated CTCs, biophenotypic epithelial/mesenchymal CTCs, and mesenchymal-dominated CTCs, according to epithelial and mesenchymal markers. Furthermore, the hormone receptor (HR) status of each CTC was determined based on the expression levels of three reference genes and was characterized by four levels, which ranged from high-level expression to non-expression. We subsequently concluded that based on EMT phenotypes, the order of different CTC subgroups differed according to the HR expression status of the primary tumor. With respect to the HR status between tissues and CTCs, the variation tendency from high-level expression to non-expression of HR in CTCs was significantly correlated with the HR status of the primary tumor. The findings could provide evidence for the potential application of this uncomplicated and practicable detection approach for prognostic analysis and individualized endocrine therapeutic direction in a real-time manner via confirmation in further large-scale trials.

## INTRODUCTION

Breast cancer is a heterogeneous group of diseases with different histological, prognostic and clinical aspects [1]. Advances in next-generation sequencing (NGS) studies have provided evidence for continuous spatial and temporal heterogeneity during tumor evolution, which is considered one of the major reasons for the current failure of cancer systemic treatments [2]. A recent technological advancement, ‘liquid biopsy,’ was achieved in the field of precision medicine involving collection of circulating tumor cells (CTCs) and circulating tumor DNA (ctDNA); it is performed at different time-points and may provide an approach for dynamic assessments of tumor characteristics. CTCs circulate in the peripheral blood stream of patients with solid malignancies. They are defined as cells meeting all of the following criteria: CD45-negative cells with a high nuclear to cytoplasmic ratio, irregular shape, hyperchromatic nuclei and diameter >10 μm [3]. The number of CTCs is used as a prognostic and pharmacodynamic biomarker with clinical utility for evaluating clinical curative effects and directing treatment decision making [4–6]. The landmark multicenter prospective trial conducted by Cristofanilli M and colleagues [7] demonstrated that the levels of CTCs, using the CellSearch System, are correlated with a reliable estimate of the disease progression and survival earlier than the estimations obtained with traditional imaging methods in metastatic breast cancer. Subsequent clinical trials have provided evidence that CTC detection is a prognostic indicator [8–10].

In addition to intratumor heterogeneity, further consideration is needed to assess the heterogeneity and clonal evolution within the CTC subpopulations. The epithelial-mesenchymal transition (EMT) has been associated with hematogenous cancer cell dissemination from the primary tumor to new organ sites, which may lead to a decrease in or loss of EpCAM expression and cannot be captured by a CellSearch-based isolation of CTCs [11]. Because CTCs are rare in peripheral blood, missing EpCAM-negative CTCs in a given patient might be the equivalent of missing all CTCs in that patient, exposing a problematic limitation of CTC-enrichment technologies that rely on affinity-based capture by exploiting the anti-EpCAM antibody [12]. Therefore, our research group has evaluated CTC classification based on the EMT phenotype in various cancers, including lung, liver, nasopharyngeal, breast, colon and gastric cancers, using the optimized CanPatrol CTC enrichment technique in a previous study. During the analysis, CTCs are classified into the following three subpopulations: epithelial-dominated CTCs (E+ CTCs), biophenotypic epithelial/mesenchymal CTCs (E+/M+ CTCs), and mesenchymal-dominated CTCs (M+ CTCs)[13].

The hormone receptor (HR) status has substantial significance in treatment decisions for both primary and metastatic breast cancers, and it may change during disease progression [14]. CTC subpopulations are also heterogeneous in terms of their estrogen and progesterone receptor (ER/PR) expression. Aktas B et al.[15] assessed the expression of the estrogen and progesterone receptors (ER/PR) in individual CTCs and demonstrated that most CTCs were ER/PR-negative despite the presence of an ER/PR-positive primary tumor. However, few studies have evaluated the ER/PR status in different CTC subpopulations of epithelial, biophenotypic epithelial/mesenchymal and mesenchymal CTCs. Thus, the ER/PR status of each CTC is further characterized based on the expression levels of three reference genes in our study.

In this study, we applied the CanPatrol CTC enrichment technique to perform an uncomplicated and practical detection approach based on the potential utility of the heterogeneity of CTCs with respect to the EMT phenotype and ER/PR status that may be applied in prognostic analysis and individualized endocrine therapeutic direction in a real-time manner. Furthermore, we investigated the relationship between the HR status of the primary tumor and different CTC subpopulations and determined the HR status of each CTC in this proof-of-principle research. This is the first example of combining the technology of enumeration in different CTC subpopulations with the assessment of the HR expression status in each CTC.

## RESULTS

### Patient characteristics

As shown in Table 1, 28 metastatic female breast cancer patients, 27 to 68 years of age, were included in the analysis. Nineteen patients were ER- and/or PR-positive, and 11 patients were HER2-positive. The majority of the included participants (82.1%) had multiple metastatic sites, and 22 participants suffered from visceral metastasis. Regarding the therapeutic setting, 13 patients underwent 1st-line treatment, and 15 patients underwent 2nd-line or more treatment.

**Table 1:** Characteristics of the recruited breast cancer patients

	patients (n)	percentage (%)
Age		
<40	6	21.4%
≥40 to <60	20	71.4%
≥60	2	7.2%
Time between first diagnosis and analysis		
≤12months	10	35.7%
> 12mongyhs	18	64.3%
HR status		
Positive	19	67.9%
Negative	9	32.1%
HER2 status		
Positive	11	39.3%
Negative	17	60.7%
Molecular subtype		
LuminalA/B	19	67.9%
Her-2 positive	5	17.9%
Triple negative	4	14.2%
Number of metastatic site		
One	5	17.9%
Multiple	23	82.1%
Position of metastatic site		
Non-visceral	6	21.4%
Visceral	22	78.6%
Therapeutic setting		
1st-line	13	46.4%
2nd-line or more	15	53.6%

### CTC isolation and characterization

**Figure 1:**
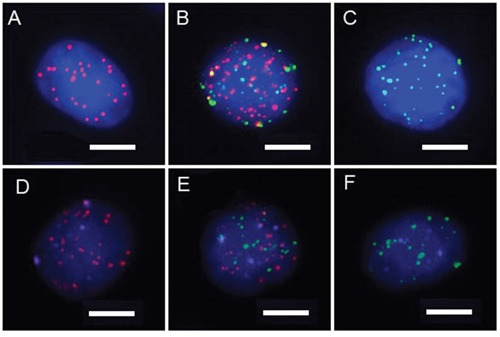
**A-C.** CTC subpopulations classified by categorical markers (A: epithelial CTCs, B: biophenotypic epithelial/mesenchymal CTCs, and C: mesenchymal CTCs). Red dots: epithelial biomarker expression. Green dots: mesenchymal biomarker expression.(Bars=5 μm). **D-F.** HR expression status of CTCs based on the expression levels of three reference genes. (D: epithelial CTCs, E: biophenotypic epithelial/mesenchymal CTCs, and F: mesenchymal CTCs). Purple dots: HR expression.(Bars=5 μm).

Each CTC was classified via categorical markers following isolation. E+ CTCs refers to the cells whose predominant markers were EpCAM and CK8/18/19; M+ CTCs mainly present with vimentin and twist markers, whereas the biophenotypic E+/M+ CTCs express both epithelial and mesenchymal markers. All blood samples obtained from the 28 included patients were identified to have CTCs (100%), as shown in Table 2. Images for each CTC subpopulation are shown in Figure 1A, 1B and 1C.

**Table 2:** Number of different CTC subpopulations in the 28 blood samples obtained from metastatic breast cancer patients

No.	Total CTCs	E+ CTCs	E+/M+ CTCs	M+CTCs
1	17	10	2	5
2	7	6	1	0
3	73	19	20	34
4	8	5	1	2
5	3	1	1	1
6	5	3	1	1
7	4	2	0	2
8	10	4	2	4
9	17	8	4	5
10	9	4	2	3
11	14	3	3	8
12	79	48	19	12
13	3	0	2	1
14	12	1	4	7
15	12	2	3	7
16	6	1	3	2
17	104	47	30	27
18	39	11	14	14
19	28	2	6	20
20	15	6	3	6
21	27	13	8	6
22	7	2	1	4
23	36	8	12	16
24	5	0	2	3
25	85	18	25	42
26	2	0	1	1
27	61	28	15	18
28	2	1	1	0

### Detection of the HR expression status of each CTC

**Figure 2:**
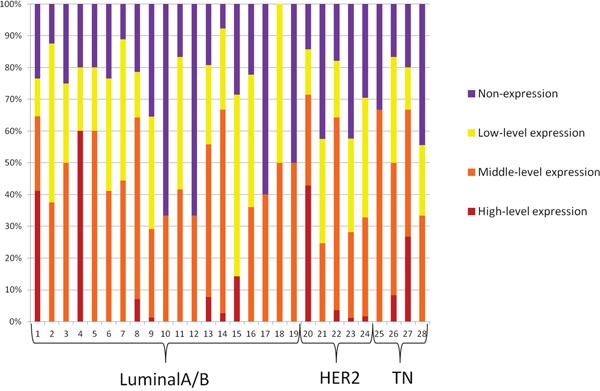
The variation tendency from high-level expression to non-expression of HR of all CTCs for each of the 28 included patients

The expression levels of the three reference genes were used to determine the HR status of each CTC. Moreover, the HR status of each CTC of the 28 included patients was characterized by four degrees, including high-level expression, middle-level expression, low-level expression and non-expression, as shown in Figure 2. Images for the HR status of each CTC subpopulation are shown in Figure 1D, 1E and 1F.

### Assessment of the CTC heterogeneity

#### Different categories of CTC subpopulations according to the different hormone receptor statuses of the primary tumor

Different categories of the CTC subpopulations of the EMT phenotype were significantly different in variation tendency from E+ CTCs to M+ CTCs between the HR-positive and HR-negative groups in the primary tumor (Z= −3.569, *P<0.001*, Table 3). Of the patients whose HR status was positive in the primary tumor, the proportion of E+ CTCs (41.7%) was increased compared with E+/M+ CTCs (27.4%) or M+ CTCs (30.9%). Regarding the samples obtained from the HR-negative patients, the M+ CTCs (43.1%) were present in a larger percentage than the E+ CTCs (30.5%) or E+/M+ CTCs (26.4%). Regarding the patients whose HR status was positive in the primary tumor, the percentage of E+ CTCs was significantly increased compared with the HR-negative patients (41.7% versus 30.5%, respectively).

**Table 3:** Different CTC subpopulations based on different hormone receptor statuses of the primary tumor

CTC subpopulations	HR status of the primary tumor		Mann-Whiteney u	P value
	Positive	Negative		
E+ CTCs	158 (41.7%)	95 (30.5%)	Z= −3.569	<0.001
E+/M+ CTCs	104 (27.4%)	82 (26.4%)		
M+ CTCs	117 (30.9%)	134 (43.1%)		

#### Different HR expression levels of CTCs based on different HR statuses of the primary tumor

Comparing the HR-positive and HR-negative groups in the primary tumor, there was a significant difference in the variation tendency of the HR expression levels of CTCs from high-level expression to non-expression (Z= −3.524, *P<0.001*, Table 4). As for the patients whose HR status was positive in the primary tumor, the percentage of high-level expression was significantly increased compared with the HR-negative patients (5.8% versus 3.5%, respectively), while the percentage of non-expression was significantly decreased compared with the HR-negative patients (23.7% versus 35.0%, respectively).

**Table 4:** Different levels of CTCs HR expression status based on different hormone receptor statuses of the primary tumors

HR status of CTCs	HR status of the primary tumor		Mann-Whiteney u	P value
	Positive	Negative		
high-level expression	22 (5.8%)	11 (3.5%)	Z= −3.524	<0.001
moderate-level expression	156 (41.2%)	101 (32.5%)		
low-level expression	111 (29.3%)	90 (28.9%)		
non-expression	90 (23.7%)	109 (35.0%)		

### Different levels of HR expression based on CTC subpopulations

For the different CTC subpopulations with the EMT phenotype, there were significantly different variation tendencies in the expression levels of the HRstatus in each CTC subpopulation (*P=0.041*, Table 5). Regarding the HR high-level expression CTCs, 8.3% were E+ CTCs, which was substantially higher than the E+/M+ CTCs (2.1%) or M+ CTCs (3.2%). The percentage of high-level expressing CTCs in E+ CTCs was approximately three times higher than for E+/M+ CTCs or M+ CTCs. As for the HR non-expression CTCs, the percentage in E+ CTCs (24.8%) is less than the E+/M+ CTCs (31.0%) or M+ CTCs (31.3%).

**Table 5:** Different levels of HR expression according to CTC subpopulation

HR status of CTCs	E+ CTCs	E+/M+ CTCs	M+ CTCs	Kruskal-Wallis H	P value
high-level expression	21 (8.3%)	4 (2.1%)	8 (3.2%)	Z=6.405	0.041
moderate-level expression	97 (38.2%)	75 (40.1%)	85 (34.1%)		
low-level expression	73 (28.7%)	50 (26.7%)	78 (31.3%)		
non-expression	63 (24.8%)	58 (31.0%)	78 (31.3%)		

## DISCUSSION

The detection of liquid biopsy at different time-points, such as CTCs and ctDNA, has been demonstrated to be a strong prognostic factor with respect to the PFS and OS in patients with metastatic breast cancer. [16–20] Bidard FC et al. [10] conducted a pooled analysis of 1944 eligible patients from 20 studies and consequently demonstrated the superiority of CTC counts in survival prediction compared with CEA and CA15-3 at each tested time point. This previous study provided level 1 evidence for the prognostic value of CTC detection at baseline and during treatment in metastatic breast cancer.

However, the heterogeneous characteristics of CTCs may limit their broad clinical application [21]. Therefore, the heterogeneity of different CTC subgroups has attracted the attention of researchers in the setting of the discovery of EMT process, which may be involved in the complicated process of tumor metastasis based on preclinical studies [22]. Königsberg R et al. [23] indicated that epithelial antigen-based approaches might fail to detect EpCAM-negative CTCs, which may have undergone the EMT process and exhibit stem cell features. Gorges TM [24] indicated that with the use of EpCAM-based detection, numerous cells escape in the blood samples of cancer patients as a result of the EMT process, which is characterized by the downregulation of epithelial markers, such as EpCAM, and upregulation of mesenchymal markers, such as Twist and EGFR, on CTCs. Hyun KA et al.[12] conducted a study on heterogeneous EpCAM expression in blood samples from breast cancer patients and provided additional evidence that decreased EpCAM expression is correlated with expression of both EMT and cancer stem cell markers.

Therefore, in our study, CTCs were labeled by both epithelial markers, EpCAM and CK8/18/19, and mesenchymal markers, vimentin and twist, making it possible to identify subpopulations based on the CTC heterogeneity. TWIST, a basic helix-loop-helix transcription factor, has been proposed as a putative biomarker for EMT [25, 26]. A positive association between the expression of TWIST in primary tumors and the risk for recurrence and poor survival has been shown in breast cancer [27–29]. Moreover, studies have reported that TWIST-expressing CTCs are frequently observed in patients with breast cancer [30, 31]. Vimentin is expressed in mesenchymal cells and is commonly considered a marker of EMT. Raimondi C, *et al.* [32] found that vimentin is expressed in CD45-/CK+ CTCs and CD45-/CK- cells, suggesting that vimentin can be used as a marker of EMT in breast cancer. Compared with the immunostaining method, this approach has the advantages of high sensitivity and background suppression.

In this study, we investigated the heterogeneity of CTC subgroups according to different HR statuses, and there were significant findings when the data were arranged in the following order of epithelial-mesenchymal transition: from E+ CTCs to E+/M+ CTCs followed by M+ CTCs. In the patients who had HR expression in the primary tumor, the proportion of E+ CTCs was increased compared with E+/M+CTCs and M+ CTCs. In the patients whose HR status was negative in the primary tumor, M+ CTCs occupied a larger percentage compared with the other two types. These findings indicate that the CTC subgroups and EMT features were related to the HR status of the primary tumor; for HR-positive breast cancer patients, E+ CTCs appear to occupy a predominant position, whereas for HR-negative patients, M+ CTCs may be dominant. This finding was consistent with a study by Yu M et al. [33] in which the CTCs from patients with ER+/PR+ primary tumors were predominantly epithelial, whereas the CTCs from the triple negative subtype (ER-/PR-/HER2-) were predominantly mesenchymal. In Yu M's study, the clinical application of M+ CTCs as a prognostic index was demonstrated by evidence that some patients who responded to therapy exhibited an increase in their CTC numbers with a proportional decrease in their M+ CTCs; meanwhile, some patients who had progressive disease during therapy exhibited a decreased number of CTCs with a proportional increase in M+ CTCs in the post-treatment sample. Hence, a heterogeneous population of CTCs could be a biomarker with better accuracy than the total CTC counts in the evaluation of therapeutic resistance and judgment of prognosis.

The decision to implement endocrine therapy for breast cancer is based on the assessment of the ER/PR status of the primary tumor immunohistochemistry in routine clinical practice, and targeting this pathway with anti-estrogen therapy has a clear clinical benefit. However, discrepancy between the HER2 and ER status of the primary tumor and metastatic lesions occurs in one-third or more of MBC patients [34]. This may explain why a proportion of HR-positive patients failed to respond to endocrine therapy. Furthermore, it may be difficult to obtain tissues for reevaluating the HR status in metastatic breast cancer because of the location of the metastatic site. Thus, evaluation of the HR status of CTCs may be an easier, more correlative approach for making metastatic breast cancer treatment decisions. Using the CanPatrol system, the HR status was evaluated based on the expression levels of three reference genes and characterized by four degrees. In our study, the variation tendency from high-level expression to non-expression of HR expression of CTCs was significantly related to the HR status of the primary tumor. The result was consistent with what Kalinsky K et al. [35] reported, which was a concordance of 68% (15/23) in the ER/PR status between primary tumors and CTCs and 83% (10/12) between metastatic tumors and CTCs. However, some researchers reported discordance in the HR status between the tissue biopsy and CTCs in primary or metastatic breast cancer. Banys M and his colleagues [36] compared the expression profiles of the primary tumor and CTC phenotype before and after removing primary tumors and demonstrated that the CTC phenotype differs from the primary tumor. Aktas B et al. [15] demonstrated discordance in the ER and PR status between the primary tumors and CTCs from metastatic breast cancer patients in 41% and 45% of cases, respectively. Controversial opinions on this topic still need to be resolved in further prospective large-scale studies. If evidence for assessing the HR status of CTCs is sufficiently reliable with future technology advancements, liquid biopsy may be used to determine the ER/PR status in treatment decision making, especially when metastatic tissue is not available or biopsy is not feasible. For example, for metastatic breast patients who have HR+ CTCs, endocrine therapy may be tried even if the HR status in the primary tumor is negative.

We also demonstrated that the variation tendency of the HR status of each CTC, arranged from high-level expression to non-expression, was statistically significant among the different CTC subpopulations. This is the first study to combine the technology of enumeration in different CTC subpopulations with the assessment of the HR expression status in each CTC in this proof-of-principle research, which may facilitate future investigations for prognostic analysis and individualized endocrine therapeutic directions in a real-time manner with this uncomplicated and practicable detection approach. However, one issue to consider is that in the exploration study for metastatic breast cancer, we compared the HR status between the primary tumor and CTCs obtained from blood samples of metastatic individuals. The times we obtained for the primary tumor and blood samples were asynchronous. Future investigations may focus on trials that compare the HR status of tumor tissues with CTCs in which the values are detected concurrently with tumor detection. This approach may illustrate the relationship between tumor and liquid biopsies and enable determination of the value of its use in decision making for endocrine therapy. Moreover, we detected the HR expression status of each CTC, whereas the HER2 status was not determined in this study. As a consequence, we will concurrently investigate the HR and HER2 statuses of each CTC in a large-scale prospective trial, which will improve the CTC subpopulation system based on EMT and its potential application in treatment decisions.

In conclusion, in this proof-of-principle study, the heterogeneity of CTCs is determined from their EMT phenotype and ER/PR status. Using the CanPatrol CTC enrichment technique, the order of different CTC subgroups differed according to the HR expression status of the primary tumor. With respect to the HR status between tissue biopsies and CTCs, the HR expression variation tendency from high-level expression to non-expression in CTCs was significantly related to the HR status of the primary tumor. Furthermore, the HR status of each CTC ranged from high-level expression to non-expression, and significant differences were identified among different CTC subpopulations. The findings could provide evidence for the potential application of this uncomplicated and practicable detection approach, allowing for prognostic analysis and individualized endocrine therapeutic directions in a real-time manner with validation in further large-scale trials.

## MATERIALS AND METHODS

### Study design

Twenty-eight metastatic breast cancer patients were recruited after an agreement from the Ethical committee between July and August in 2015 from Cancer Hospital, Chinese Academy of Medical Sciences and Peking Union Medical College. The patient characteristics, such as the immunohistochemical phenotype of the primary tumor and metastatic sites, were collected. Five milliliters of peripheral blood samples (anticoagulated with EDTA) was collected after discarding the initial 2 ml to avoid potential skin cell contamination from the venipuncture site; the samples were stored at 4°C for further analysis. Blood was drawn before the start of a new therapy type.

### Patient selection

The major inclusion criteria were as follows: female patients aged ≥18 years with histologically confirmed primary breast cancer who were diagnosed with distant metastatic disease. The sample series includes breast cancer patients with different molecular pathology features. All patients signed informed consent for the use of their blood samples. Prior adjuvant treatment, radiation or any other treatment for metastatic disease was permitted. However, secondary primary malignancies were excluded.

### Isolation and classification of CTCs using the Canpatrol system

CTC isolation was conducted using the Canpatrol CTC filtration system, whichincluded a filtration tube (SurExam, Guangzhou, China) that contained a calibrated membrane with 8-μm diameter pores (SurExam, Guangzhou, China), a manifold vacuum plate with valve settings (Millipore, Billerica, USA), an E-Z 96 vacuum manifold (Omega, Norcross, USA), and a vacuum pump (Auto Science, Tianjin, China). Prior to filtration, red blood cell lysis buffer (154 mM NH_4_Cl, 10 mM KHCO_3_ and 0.1 mM EDTA in deionized water, Sigma, St. Louis, USA) was applied to remove the erythrocytes. PBS with 4% formaldehyde (Sigma, St. Louis, USA) was subsequently used to resuspend the remaining cells. The cell suspension was transferred to a filtration tube and pumped with at least 0.08 MPa. The membrane with isolated CTCs was ultimately obtained.

CTCs were classified using a multiplex RNA-*in situ* hybridization (RNA-ISH) assay. Four epithelial (E) biomarkers (EpCAM and CK8/18/19), two mesenchymal (M) biomarkers (vimentin and twist) and a leukocyte biomarker, CD45, were used to capture and characterize the CTCs. A detailed hybridization assay was performed as previously described [13]. The assay was performed in a 24-well plate (Corning, NY, USA), and the cells on the membrane were treated with a protease (Qiagen, Hilden, Germany) and subsequently subjected to serial hybridization reactions with capture probes that were specific for the intended examined genes, as previously described. Three types of fluorescently labeled probes were added and incubated. The sequences of the capture probes and bDNA signal amplification probes have been previously published [13] and were synthesized by Invitrogen (Invitrogen, Shanghai, China). The cell nuclei were stained with 4′, 6-diamidino-2-phenylindole (DAPI) (Sigma, St. Louis, USA) and analyzed with an automatic fluorescence microscope Axio Imager Z2 (Zeiss, Carl Zeiss Meditec AG, Germany). The red and green dots of the fluorescent signal represent the epithelial and mesenchymal biomarker expression, respectively. The white fluorescent dots represent CD45 gene expression.

### Detection of the HR status of each CTC

The HR (ER and PR) expression level was divided into four degrees, including non-expression, low-level, middle-level and high-level, based on the expression levels of the following three reference genes: TBP (encodes TATA box-binding protein), TFRC (transferrin receptor) and B2M (microglobulinbeta-2). These genes exhibited different expression abundances, namely low-level, middle-level and high-level, in the CTCs. The fluorescence intensities of the three reference genes in 100 clinical CTC samples were independently calculated, and the corresponding cut-off values were defined as 5.5 and 12.5 according to the ROC curves. ER and PR were detected with hybridization and labeled using Alexa Fluor 647 (purple fluorescent dots). The capture probe sequences for ER and PR and the sequences for the bDNA signal amplification probes are shown in Supplementary Tables 1 and 2.

### Statistical analysis

Statistical analysis was performed using SPSS, version 22.0 (SPSS Inc., Chicago, IL, USA). The results are presented as percentages for categorical variables. The Mann-Whitney U test was used to assess the differences between two groups because the data were not normally distributed, and the Kruskal-Wallis H test was used for multi-group analysis. P values less than 0.05 were considered statistically significant.

## SUPPLEMENTARY MATERIALS TABLES


