# Supplementary material for: Analysis of the hormone receptor status of circulating tumor cell subpopulations based on epithelial-mesenchymal transition: a proof-of-principle study on the heterogeneity of circulating tumor cells
Source: Oncotarget. 2016 Sep 1;7(40):65993–6002. doi: 10.18632/oncotarget.11787 (PMC5323209; doi:10.18632/oncotarget.11787)
Supplement: Supplementary file 1 [file oncotarget-07-65993-s001.pdf]

Analysis of the hormone receptor status of circulating tumor cell subpopulations based on epithelial-mesenchymal transition: a proof-of-principle study on the heterogeneity of circulating tumor cells

SUPPLEMENTARY TABLES

Supplementary Table S1: Capture probe sequences for ER and PR genes

Gene	Sequences (5’→3’)
ER	GAAAAAGAGCACAGCCCGAG
	CGCTTGTGTTTCAACATTCT
	GACGAGACCAATCATCAGGA
	TGCAGATTCATCATGCGGAA
	TTGGCTAAAGTGGTGCATGA
	AGCAAATGAATGGCCACTCA
PR	GTTCTCATTGAGAATGCCAC
	TGCGACGGCAATTTAGTGAC
	TTGTTAGGAGATCTCGTCTC
	CGAAACTTCAGGCAAGGTGT
	TAGGCACGTGGATGAAATCC
	GACTCGAAGCTGTATTGTGG

Supplementary Table S2: bDNA signal amplification probes sequences for ER and PR genes

	Function (copies)	Sequences (5'→3')	Complement
bDNA probes for ER and PR	capture probe tail(1)	AATCCTTTCTTTAATCTCAAATCAAATCTACAAATCCAATAATCTCAT	preamplifier leader(1)
	preamplifier repeat(5)	ATGAGATTATTGGATTTGTAGATTATTATTGAGATGTGAAGTTTGTTT	amplifier leader(1)
	amplifier repeat(5)	AAACAAACTTCACATCTCAATAAT	label probe(1)

The sequences labeled "leader" appear once in the indicated construct, while sequences labeled "repeat" appear the indicated number of times. The tail on the capture probe is a single sequence.
